# Continuous infusion [^11^C]-(+)-PHNO PET for measuring dopamine D_2/3_ receptor availability and amphetamine effects across the brain: Feasibility and test-retest reliability

**DOI:** 10.1007/s00259-026-07998-w

**Published:** 2026-06-15

**Authors:** Irena Dajic, Ulrich Sauerzopf, Ana Weidenauer, Cornelia Diendorfer, Katharina Landesmann, Ivo Rausch, Andreas Hahn, Benjamin Spurny-Dworak, Manfred Klöbl, Murray Reed, Rupert Lanzenberger, Martin Bauer, Markus Zeitlinger, Lucie Bartova, Martin Wolkersdorfer, Lukas Nics, Wolfgang Wadsak, Marcus Hacker, Cécile Philippe, Nicole Praschak-Rieder, Matthäus Willeit

**Affiliations:** 1 Department of Psychiatry and Psychotherapy, Division of General Psychiatry, Medical University of Vienna, Vienna, Austria; 2 Comprehensive Center for Clinical Neurosciences and Mental Health, Medical University of Vienna, Vienna, Austria; 3 QIMP Team, Center for Medical Physics and Biomedical Engineering, Medical University of Vienna, Vienna, Austria; 4 Department of Clinical Pharmacology, Medical University of Vienna, Vienna, Austria; 5 Psychosocial Services Vienna, Vienna, Austria; 6 Department of Production, Landesapotheke Salzburg, Hospital Pharmacy, Salzburg, Austria; 7 Department of Biomedical Imaging and Image Guided Therapy, Medical University of Vienna, Vienna, Austria

**Keywords:** Bolus constant infusion, Cerebellum, Cortical dopamine, Dopamine release, Positron emission tomography

## Abstract

**Purpose:**

Dopamine D_2/3_ receptors are expressed throughout the human brain, but synchronous receptor quantification in subcortical and cortical brain areas remains difficult to achieve with a single radioligand. Here, we assessed the suitability of a bolus plus constant infusion (B/I) paradigm for brain-wide dopamine D_2/3_ receptor imaging with the agonist radioligand [^11^C]-(+)-PHNO.

**Methods:**

Five healthy male volunteers underwent two B/I [^11^C]-(+)-PHNO positron emission tomography (PET) scans combined with a mid-scan intravenous amphetamine challenge. Non-displaceable binding potentials (BP_ND_) were calculated using the equilibrium ratio method and an algorithmically constrained cerebellum as the reference region.

**Results:**

Reproducible baseline BP_ND_ values were obtained for the majority of regions examined, with intraclass correlation coefficients ranging from 0.5 to 0.93, and test-retest variability remaining below 10%. Moreover, amphetamine-induced decreases in receptor binding were observed in both scans in cortical and a number of subcortical regions. In the globus pallidus and ventral striatum, however, equilibrium had not been reached prior to amphetamine injection, suggesting that further optimization of the protocol might be needed to quantify amphetamine effects in D_3_ receptor-rich regions.

**Conclusions:**

These results indicate that, within certain limitations, synchronous cortical and subcortical measurements of D_2/3_ receptors at baseline and under challenge conditions can be achieved using the B/I approach and [^11^C]-(+)-PHNO PET.

**Supplementary information:**

The online version contains supplementary material available at https://doi.org/10.1007/s00259-026-07998-w.

## Introduction

Dysregulated dopamine neurotransmission underlies a wide range of neurological and psychiatric conditions, including schizophrenia, attention deficit hyperactivity disorder, Parkinson’s disease, and substance use disorders. Studying the dopamine system in vivo using single photon emission computed tomography (SPECT) and positron emission tomography (PET) has helped us make rapid strides in characterizing the dynamics of neurotransmitter signalling and their alterations in these disorders [1]. Our understanding of the relevant processes, however, remains limited, due in part to methodological constraints, one of which is the difficulty of synchronously measuring dopamine activity in brain areas with widely varied dopaminergic innervation.

Dopamine D_2/3_ receptors are found throughout the human brain, but their expression patterns display vast regional variability, with an order of magnitude differences between the densely populated subcortical regions and receptor-sparse cortical areas [2, 3]. As radioligands most suitable for imaging low receptor concentrations typically have high affinities for their targets, and their binding in densely populated areas consequently requires lengthy intervals to estimate, these differences in expression present a considerable obstacle to synchronous estimation of D_2/3_ receptor availability with a single radioligand [4].

The aim of the present study was to assess the feasibility of simultaneous cortical and subcortical dopamine D_2/3_ receptor quantification at baseline and under challenge conditions using a single-scan bolus plus constant infusion (B/I) paradigm with [^11^C]-(+)-PHNO PET [5–7].

[^11^C]-(+)-PHNO, a D_3_-preferring dopamine D_2/3_ receptor agonist, is highly sensitive to fluctuations in endogenous neurotransmitter concentrations, as demonstrated in studies using the catecholamine-releasing compound dextro-amphetamine [8–11]. Moreover, its binding has been shown to reflect extracellular dopamine concentrations even at baseline [12].

Continued administration of a radioligand throughout the scan ideally achieves stable concentrations in tissue and blood, allowing a constant level of binding to be maintained over a period of time. Given this approximation to true equilibrium conditions, specific binding can be quantified as a simple ratio of radioactivity measured in a region of interest to that in plasma or a reference region [13–15]. By obviating the need for kinetic modelling and, at least in principle, allowing for shorter scan times, the B/I paradigm enables within-scan interventions that would otherwise entail multiple imaging sessions or complex quantification strategies. Furthermore, sustained concentrations of the tracer in tissue combined with model-free quantification might be particularly advantageous for imaging areas with lower receptor densities [16], where binding potentials are in many cases difficult to estimate with a single bolus injection approach [4].

The B/I approach has been successfully used to quantify amphetamine-induced dopamine release in the striatum and some extrastriatal regions with D_2/3_ receptor antagonist radioligands [^123^I]Iodobenzamide [17, 18], [^11^C]Raclopride [19–21], and [^18^F]Fallypride [22]. More recently, the paradigm has been employed in estimating subcortical dopamine D_2/3_ receptor availability with [^11^C]-(+)-PHNO [7, 23].

To extend these results, and assess the suitability of the B/I paradigm for detecting changes in extracellular dopamine concentrations in the cortex as well as subcortical areas, we performed test-retest B/I [^11^C]-(+)-PHNO PET scans coupled with mid-scan intravenous amphetamine challenges in five healthy volunteers.

## Methods

### Subjects and study procedures

The study took place at the Medical University of Vienna and was approved by the local Ethics Committee (EK 1148/2017). After a full explanation of study procedures and risks, all participants provided written informed consent.

Five healthy male volunteers, aged 24 to 38 years, were recruited via local advertisements and word of mouth. Screening procedures included physical examination, routine laboratory analyses, and an electrocardiogram to ensure good general health. Absence of a psychiatric disorder was ascertained through a structured clinical interview for DSM-IV diagnoses (M.I.N.I.) [24]. Urine drug tests (for amphetamines, benzodiazepines, opiates, methadone, cocaine, PCP, and tricyclic antidepressants) were performed at inclusion and every subsequent study visit. Each volunteer underwent two B/I [^11^C]-(+)-PHNO PET scans combined with an amphetamine challenge, approximately one week apart. To aid PET image analysis, a 3D T1-weighted MPRAGE (1mm^3^ voxel size, TR = 2300 ms, TI = 900 ms, TE = 2.25 ms, flip angle = 8°) magnetic resonance (MR) scan was acquired on a Siemens MAGNETOM Prisma 3T scanner (Siemens Healthineers, Erlangen, Germany).

### Radiochemistry

[^11^C]-(+)-PHNO was synthesized as described previously [5, 25, 26]. At the end of production, molar activity was 132.54 ± 80.1 GBq/µmol and 127.74 ± 44.31 GBq/µmol for scans one and two, respectively. There were no significant differences between the first and the second scan in terms of applied dose (scan one: 389.95 ± 96.06 MBq, scan two: 441.96 ± 103.08 MBq; two-tailed t=-0.8, *p* = 0.43), mass (scan one: 1.24 ± 0.56 µg, scan two: 1.23 ± 0.27 µg; t = 0.06, *p* = 0.94) or molar activity (scan one: 96.2 ± 60.88 GBq/µmol, scan two: 93.6 ± 33.93 GBq/µmol; t = 0.13, *p* = 0.9), although the variability of injected mass and molar activity were noticeably higher in scan one.

### Positron emission tomography

PET data were acquired on a GE Advance scanner (General Electric Medical Systems, Milwaukee, WI) with a spatial resolution of 6 mm full-width at half-maximum (FWHM). Following a short transmission scan, performed using a Ge^68^ rotating source for the purposes of attenuation correction, emission data were recorded over 120 min starting with the tracer injection. [^11^C]-(+)-PHNO was applied as a bolus over one minute followed by constant infusion with a kbol (bolus portion in relation to equivalent infusion duration) of 80 min. After 60 min of scanning, dextro-amphetamine sulfate at a dose of 0.3 mg/kg was administered intravenously in 100 ml isotonic saline solution over 2 to 3 min. Scanning continued for another 60 min (with the exception of one scan, which was ended 20 min early).

### Image pre-processing

Dynamic PET data were reconstructed using filtered-back projection (pixel size 3.125 × 3.125 × 4.250 mm) into 36 frames (15 1-minute frames followed by 21 5-minute frames). The resulting images were corrected for attenuation, scatter, and decay. Further image processing steps were carried out in AFNI (version Marcus Aurelius) [27]. Frame-wise motion correction to an averaged sequence of early long frames (15–40 min of scanning) was performed using a weighted least squares algorithm and Fourier interpolation. PET images were co-registered to the subjects’ T1-weighted MR scans using normalized mutual information as the cost function, and non-linearly aligned to a high-resolution MR image template in Montreal Neurological Institute (MNI) space [28]. All subsequent image analyses were performed in MNI space. Please see Supplementary Material (**Table ****S1**) for details on the pre-processing pipeline.

### Regions of interest

Regions of interest (ROIs) were delineated on participants’ MR images using Freesurfer’s (version 6.0) [29] automated pipeline with reference to the Destrieux atlas [30], and comprised: caudate, putamen, ventral striatum, globus pallidus, thalamus, insula, hippocampus, amygdala, anterior cingulate cortex, inferior frontal gyrus, orbitofrontal cortex, dorsolateral prefrontal cortex, superior, middle and inferior temporal gyrus. In addition, a combined ROI for substantia nigra and ventral tegmental area (SN/VTA) was created using the extended Human Connectome Project (HCP) atlas [31]. These regions were selected for consistency with relevant PET studies and post-mortem data on dopamine D_2/3_ receptor expression in the human brain [3, 32]. Bilateral time activity curves (TACs) for each ROI were obtained by averaging activities from left and right hemispheres. Atlas regions used in the creation of composite ROIs are listed in the Supplement (**Table** S4).

### Reference region

In line with common practice, the cerebellum was chosen as the reference region, as it contains low levels of dopamine D_2/3_ receptors [33, 34]. However, given concerns about its suitability for accurate determination of non-displaceable uptake (combined free and non-specifically bound fraction) when using [^11^C]-(+)-PHNO and other dopamine D_2/3_ radioligands [35–39], we restricted the reference region to the approximately 50% of cerebellar gray matter voxels with the lowest radioligand uptake during the pre-amphetamine equilibrium. Details on the thresholding procedure and anatomical overlap are reported in the Supplement.

### Equilibrium analysis

Accurate quantification with the B/I paradigm essentially depends on performing measurements at, or in close proximity to equilibrium conditions. Under ideal circumstances, this would be ensured by optimizing the infusion schedule for each volunteer using parameters obtained in a prior bolus experiment [40]. A more practical alternative is to define a group-wise infusion protocol likely to achieve equilibrium during the desired scanning interval, as estimated from existing data on radioligand kinetics [15]. Based on a previous B/I [^11^C]-(+)-PHNO PET study using the same bolus to infusion ratio [7], tracer equilibrium was expected to occur between 30 and 60 min after bolus injection in most ROIs. To accommodate any individual differences, which in the case of [^11^C]-(+)-PHNO may include the effects of mass [10, 35], we allowed equilibrium time windows to vary in placement and length within the following scanning intervals: 30- and 60-min following tracer injection for baseline equilibrium (3–6 consecutive frames) and 60–120 min of scanning for the post-amphetamine equilibrium (3–8 consecutive frames). The greater length of the post-amphetamine scanning period afforded greater flexibility, which in turn helped mitigate the effects of noise characteristic for later stages of B/I studies. Specific time windows were selected based on the rates of change in measured activity - a common metric for evaluating equilibrium in B/I experiments - calculated for voxels with high [^11^C]-(+)-PHNO uptake. As the goal of the study was to assess the feasibility of acquiring synchronous receptor binding estimates across the brain, this approach was designed to select the best overall time point for quantification, only slightly favouring regions with higher receptor density (see Supplementary Material for exact parameters and resulting equilibrium placement).

Non-displaceable binding potentials (BP_ND_) [41] were calculated as the equilibrium ratio of radioactivity concentrations in target regions and the reference region, i.e. the thresholded cerebellum:

$$\:{BP}_{ND}=\frac{{C}_{ROI}}{{C}_{tCER}}-1$$

The equilibrium ratio method was also employed to determine BP_ND_ values at a single voxel level.

### Statistical analysis

Statistical analysis was performed in R version 3.6.0 (R Foundation for Statistical Computing, Vienna, Austria; https://www.r-project.org). Voxel-based analyses were carried out using functions implemented in AFNI. Test-retest reliability of regional BP_ND_ values at baseline was evaluated with intraclass correlation coefficients (ICC 3,1) [42] reported according to Koo and Li criteria [43]. Parallel analyses were performed at voxel level using AFNI’s 3dICC program [44]. Consistency, rather than absolute agreement, was chosen as the outcome of interest in order to account for any effects of repeated amphetamine administration [45–47]. Relative and absolute test-retest variability (TRV and aTRV) were calculated as percent change between first and second scan:

$$\:TRV=\frac{2\left(Test-Retest\right)}{Test+Retest}*100$$

$$\:aTRV=\frac{\left|2\left(Test-Retest\right)\right|}{Test+Retest}*100$$

Post-amphetamine changes in [^11^C]-(+)-PHNO binding were expressed as percent difference between baseline and post-amphetamine BP_ND_ values (henceforth BP_ND−BASE_ and BP_ND−AMPH_).

$$\:\varDelta\:{BP}_{ND}=\frac{\left({BP}_{ND-BASE}-{BP}_{ND-AMPH}\right)}{{BP}_{ND-BASE}}*100$$

BP_ND−BASE_ and BP_ND−AMPH_ were compared separately for the first and second scan by means of two-tailed paired sample t-tests. Differences between first and second scan BP_ND_ values were likewise assessed.

Given the small sample size and the pilot aspects of the study, no correction for multiple comparisons was performed and all p values < 0.05 are reported. Brain images were rendered in AFNI and labelled in Inkscape version 1.4 (Inkscape Project, 2024; https://inkscape.org). All other figures were created using R.

## Results

**Fig. 1:**
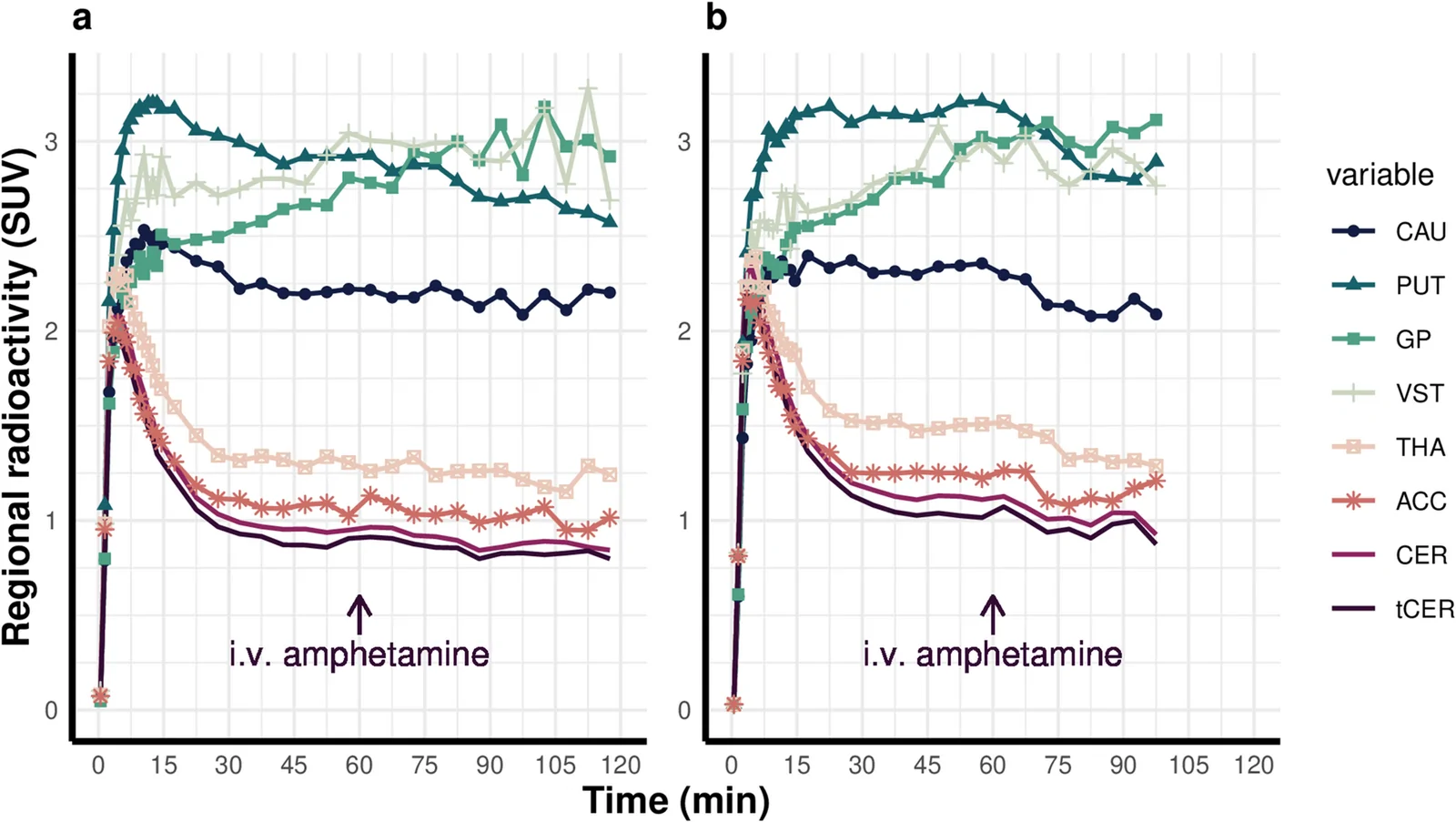
Regional time activity curves (TACs) from a 120-minute (**A**) and a 100-minute [^11^C]-(+)-PHNO PET scan (**B**) SUV: standard uptake values; CAU: caudate; PUT: putamen; GP: globus pallidus; VST: ventral striatum; THA: thalamus; ACC: anterior cingulate cortex; CER: cerebellum; tCER: thresholded cerebellum

Sample TACs are shown in **Fig.** 1. Tissue radioactivity concentrations reached stable levels within 45 min of scanning in most ROIs; the main exceptions were the ventral striatum and globus pallidus, where similarly to previous reports, TACs retained an upward slope [7]. In the majority of other regions, the rates of change during the pre-amphetamine equilibrium remained below 10% (**Table **S2). Shortly after the amphetamine injection, there was a noticeable drop in measured radioactivity in a number of cortical as well as subcortical ROIs. [^11^C]-(+)-PHNO equilibrium binding was re-established within 30 min of amphetamine injection. In ROIs where equilibrium had not been reached prior to amphetamine administration, we observed a change in the slope of the TACs (**Table **S2).

### Reference region

**Fig. 2:**
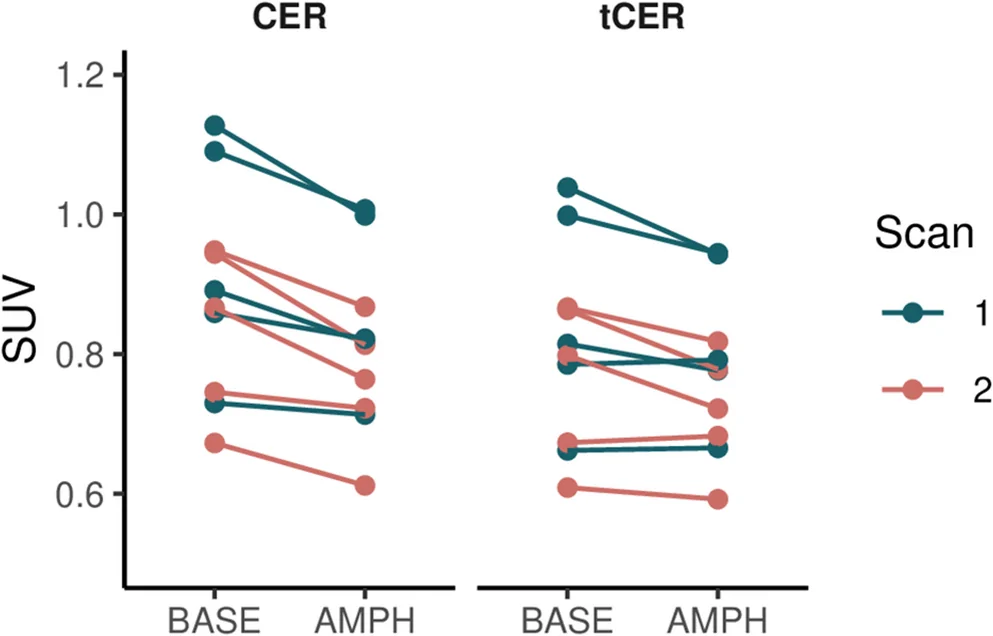
[^11^C]-(+)-PHNO standard uptake values (SUV) before (BASE) and after intravenous amphetamine (AMPH) in the unthresholded (CER) and thresholded (tCER) cerebellar cortex

Amphetamine effects were also evident in the cerebellum but were considerably less pronounced within its thresholded part (**Fig.** 2). Uptake-based thresholding resulted in reference region masks that showed a within-subject anatomical overlap of 70%; voxels that survived the thresholding procedure were most consistently located in the dorsal parts of the cerebellar cortex (**Fig. **S2). This pattern coincides with findings of a blocking study which identified [^11^C]-(+)-PHNO binding in this section of the cerebellum as least vulnerable to dopamine D_3_ receptor antagonists [48]. At baseline, SUVs of the tCER closely matched those of the unthresholded cerebellum after amphetamine administration. Amphetamine effects were statistically significant only in the unthresholded cerebellum where the change in SUV from the pre- to the post-amphetamine equilibrium extended up to 14% (**Table** 1).

**Table 1:** Amphetamine effects on standard uptake values in the thresholded and unthresholded cerebellum

	Scan	SUV_−BASE_	SUV_−AMPH_	Δ SUV (%)	*p*
CER	1	0.94 ± 0.16	0.87 ± 0.13	6.78 ± 3.57	0.02
	2	0.83 ± 0.12	0.75 ± 0.1	9.26 ± 4.07	0.01
tCER	1	0.86 ± 0.16	0.82 ± 0.12	3.57 ± 4.25	0.13
	2	0.76 ± 0.12	0.72 ± 0.09	5.27 ± 4.75	0.07

### Baseline [^11^C]-(+)-PHNO binding

At baseline, mean BP_ND_ values in the striatum and extrastriatal subcortical regions were within 10% of estimates derived using a single bolus approach and the same PET scanning system [49] (**Table **S3). Radioligand binding outside of the basal ganglia paralleled dopamine D_2/3_ receptor gradients described in multiple autoradiography and PET studies (see for example [34, 50, 51]), with highest receptor binding in the thalamus and the amygdala, followed by the insular and anterior cingulate cortex and temporal lobe structures (**Fig. **S3). Lowest binding was observed in the prefrontal cortex. Both baseline and post-amphetamine BP_ND_ tended toward higher values in the second scan, although between scan difference reached significance only in the VST (BP_ND-BASE_ t=-4.05, *p* = 0.01; BP_ND-AMPH_ t=-3.33, *p* = 0.03) and the GP (BP_ND-BASE_ t=-2.94, *p* = 0.04) These changes parallel the increase in baseline BP_ND_ in the two regions seen after exposure to multiple doses of oral amphetamine [49], thus raising the possibility of slight sensitization effects.

Intraclass correlation coefficients for BP_ND-BASE_ were excellent (> 0.9) for the striatal ROIs, hippocampus, insula, and the SN/VTA, and ranged from moderate (0.5–0.75) to good (0.75–0.9) for the anterior cingulate cortex, inferior frontal and temporal gyri, amygdala, thalamus, and the precuneus. In the remaining ROIs, i.e. superior and middle temporal gyri, orbitofrontal, and dorsolateral prefrontal cortex, group means were reproduced in the second scan, but the rank order of individual BP_ND_ values was not preserved and consequently ICC values were poor. This may have been in part due to suboptimal ROI delineation, as the outcomes for the Destrieux and HCP atlas subregions showed considerable heterogeneity (**Table **S4). Mean TRV was low overall, with all regions but the VST and orbitofrontal cortex remaining below 10%, though cortical regions in general displayed much higher variability than subcortical ROIs; aTRV remained below 20% in all regions except the orbitofrontal and dorsolateral prefrontal cortex (**Table** 2).

**Table 2:** [^11^C]-(+)-PHNO B/I binding potential values and associated statistics

	Scan	BP_ND−BASE_	BP_ND−AMPH_	Δ BP_ND_ (%)	*p*	Test - retest (BP_ND−BASE_)		
Subcortical regions						ICC	TRV (%)	aTRV (%)
CAU	1	1.48 ± 0.18	1.39 ± 0.13	5.67 ± 5.9	0.11	0.93	-4.78 ± 4.9	5.17 ± 4.3
	2	1.56 ± 0.26	1.47 ± 0.29	6.6 ± 7.5	0.11			
PUT	1	2.32 ± 0.22	2.1 ± 0.11	9.08 ± 8	0.08	0.93	-2.84 ± 4.3	4.29 ± 2.3
	2	2.39 ± 0.29	2.25 ± 0.3	6.11 ± 3.5	0.02			
VST^NE^	1	2.05 ± 0.28	2.22 ± 0.37	-8.34 ± 6.6	0.05	0.92	-10.02 ± 5.6	10.02 ± 5.6
	2	2.27 ± 0.32	2.5 ± 0.42	-10.14 ± 7.2	0.04			
GP^NE^	1	1.98 ± 0.17	2.36 ± 0.27	-19.5 ± 11.9	0.02	0.92	-4.42 ± 3.6	4.42 ± 3.6
	2	2.06 ± 0.16	2.4 ± 0.3	-16.23 ± 9.6	0.02			
SN/VTA^NE^	1	0.83 ± 0.15	1.06 ± 0.15	-28.33 ± 10.9	0.003	0.91	-8.61 ± 6.5	9.3 ± 5.1
	2	0.9 ± 0.12	1.07 ± 0.16	-18.62 ± 15.1	0.04			
THA	1	0.51 ± 0.04	0.49 ± 0.06	3.87 ± 8.2	0.36	0.75	-0.65 ± 6.3	4.38 ± 4.1
	2	0.51 ± 0.04	0.49 ± 0.06	3.97 ± 8.2	0.33			
AMY	1	0.41 ± 0.07	0.36 + 0.1	10.87 ± 25.4	0.37	0.67	-6.59 ± 12.7	11.03 ± 7.9
	2	0.44 ± 0.07	0.38 ± 0.11	15.17 ± 15.2	0.07			
Cortical regions								
INS	1	0.36 ± 0.09	0.26 ± 0.04	26 ± 11.6	0.02	0.9	-4.44 ± 8.7	8.0 ± 4.5
	2	0.38 ± 0.08	0.3 ± 0.06	20.16 ± 6	0.002			
ACC	1	0.23 ± 0.03	0.16 ± 0.03	27.48 ± 18.2	0.03	0.62	-4.23 ± 10.3	9.01 ± 5.1
	2	0.24 ± 0.02	0.18 ± 0.04	23.22 ± 16.2	0.03			
HIP	1	0.22 ± 0.04	0.17 ± 0.05	23.08 ± 26.1	0.11	0.92	-0.39 ± 8.5	6.84 ± 3.7
	2	0.22 ± 0.04	0.18 ± 0.04	18.31 ± 17	0.06			
ITG	1	0.15 ± 0.02	0.1 ± 0.03	35.14 ± 20.6	0.02	0.52	-4.65 ± 16.4	11.98 ± 10.8
	2	0.16 ± 0.03	0.12 ± 0.03	25.27 ± 9.9	0.01			
MTG	1	0.17 ± 0.03	0.11 ± 0.04	34.96 ± 25.3	0.04	0.06	-6.16 ± 19.7	15.99 ± 10.8
	2	0.18 ± 0.01	0.13 ± 0.02	28.42 ± 12.2	0.01			
STG	1	0.15 ± 0.03	0.1 ± 0.03	28.8 ± 22.1	0.09	0.34	-9.53 ± 19.7	17.78 ± 10.3
	2	0.16 ± 0.02	0.11 ± 0.02	28.3 ± 15.7	0.02			
PREC	1	0.16 ± 0.04	0.13 ± 0.04	15.28 ± 14	0.07	0.77	-2.72 ± 15.4	12.75 ± 6.6
	2	0.16 ± 0.03	0.13 ± 0.02	18.84 ± 10.2	0.02			
IFG	1	0.12 ± 0.05	0.06 ± 0.02	44.81 ± 27	0.06	0.72	-4.52 ± 23.7	19.19 ± 11.2
	2	0.12 ± 0.03	0.07 ± 0.03	40.9 ± 13.6	0.002			
OFC	1	0.12 ± 0.03	0.08 ± 0.02	29.62 ± 32.1	0.12	0.08	-11.53 ± 31.8	28.33 ± 13.2
	2	0.13 ± 0.03	0.1 ± 0.03	22.34 ± 16.4	0.03			
DLPFC	1	0.11 ± 0.04	0.06 ± 0.03	55.62 ± 32.1	0.01	0.00	-7.09 ± 39.3	28.32 ± 24.5

*BP*_*ND-BASE*_ baseline binding potential values, *BP*_*ND-AMPH*_ post-amphetamine binding potential values,* Δ BP*_*ND*_ percent change in BP_ND_ values, *p* *p* values of two-tailed paired sample t-tests, *ICC *intraclass correlation coefficient, *TRV* relative test retest variability, *aTRV* absolute test retest variability, *CAU* caudate, *PUT *putamen, *VST* ventral striatum, *GP* globus pallidus, *SN/VTA* substantia nigra/ventral tegmental area, *THA* thalamus, *AMY* amygdala, *INS *insula, *ACC* anterior cingulate cortex, *HIP* hippocampus, *ITG* inferior temporal gyrus, *MTG *middle temporal gyrus, *STG* superior temporal gyrus, *PREC* precuneus, *IFG* inferior frontal gyrus, *OFC *orbitofrontal cortex, *DLPFC *dorsolateral prefrontal cortex, NE not at equilibrium

**Fig. 3:**
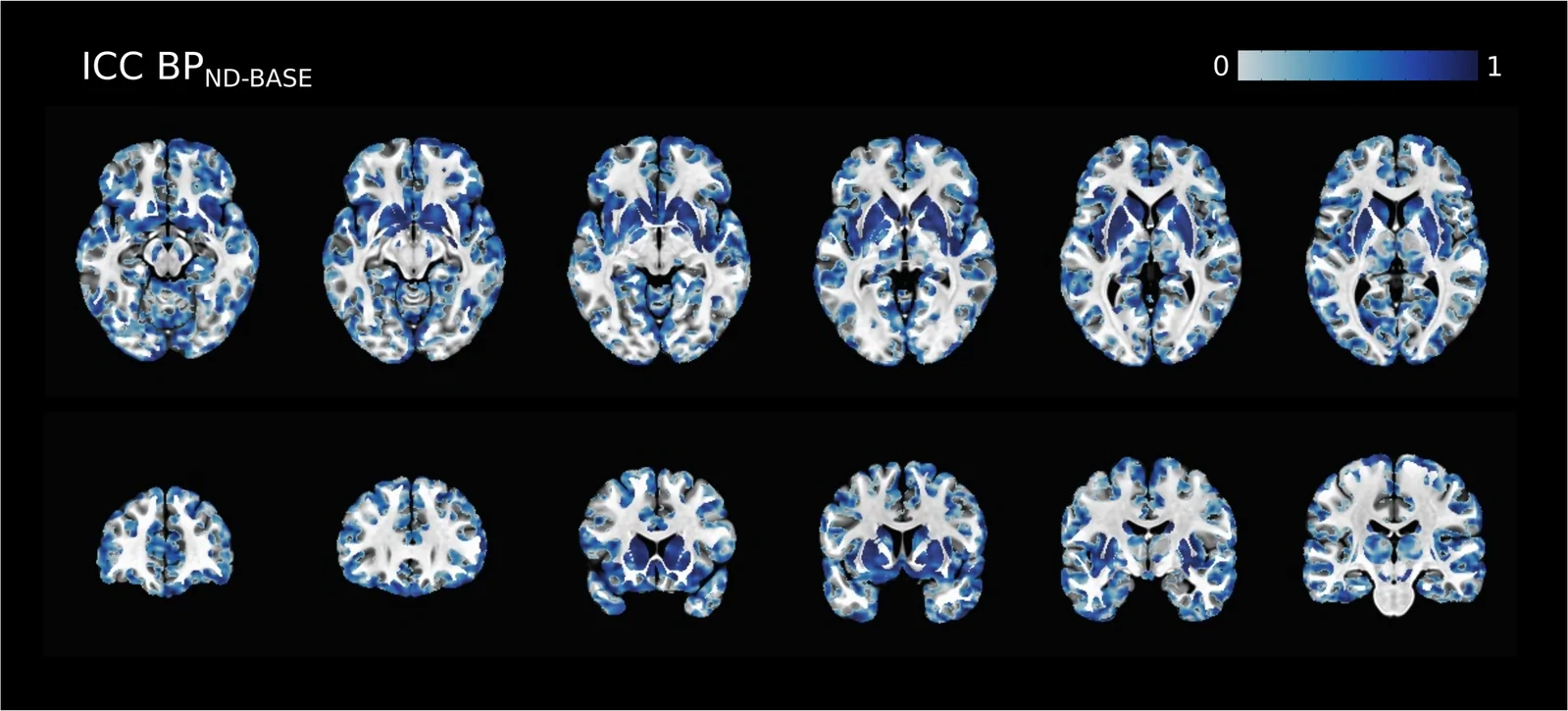
Test-retest reliability (intraclass correlation coefficients; ICC) of baseline [^11^C]-(+)-PHNO BP_ND_ values at single-voxel level (MNI coordinates z = -16 to 4, y = 44 to – 21, left is left; white matter masked out)

Whole brain analyses yielded largely concordant results, with high ICCs (**Fig.** 3) and low variability across a substantial portion of the brain even at a single voxel level. Notably, this included some of the areas where ROI-based analyses indicated low reproducibility.

### Amphetamine effects

**Fig. 4:**
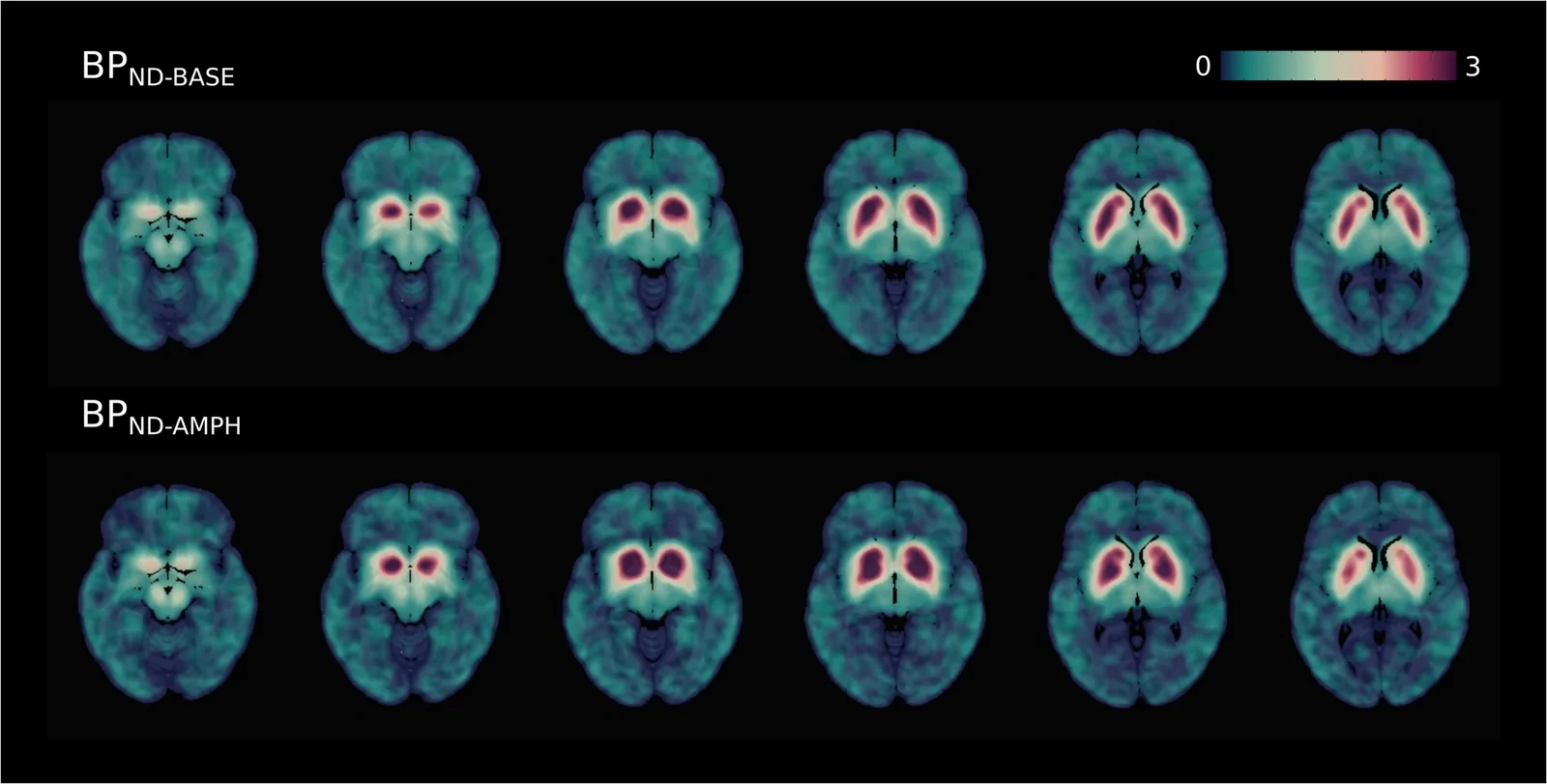
Mean [^11^C]-(+)-PHNO BP_ND_ values before (BP_ND−BASE_) and after (BP_ND−AMPH_) amphetamine administration (first scan only, *n* = 5; MNI coordinates z = -15 to 5, left is left)

**Fig. 5:**
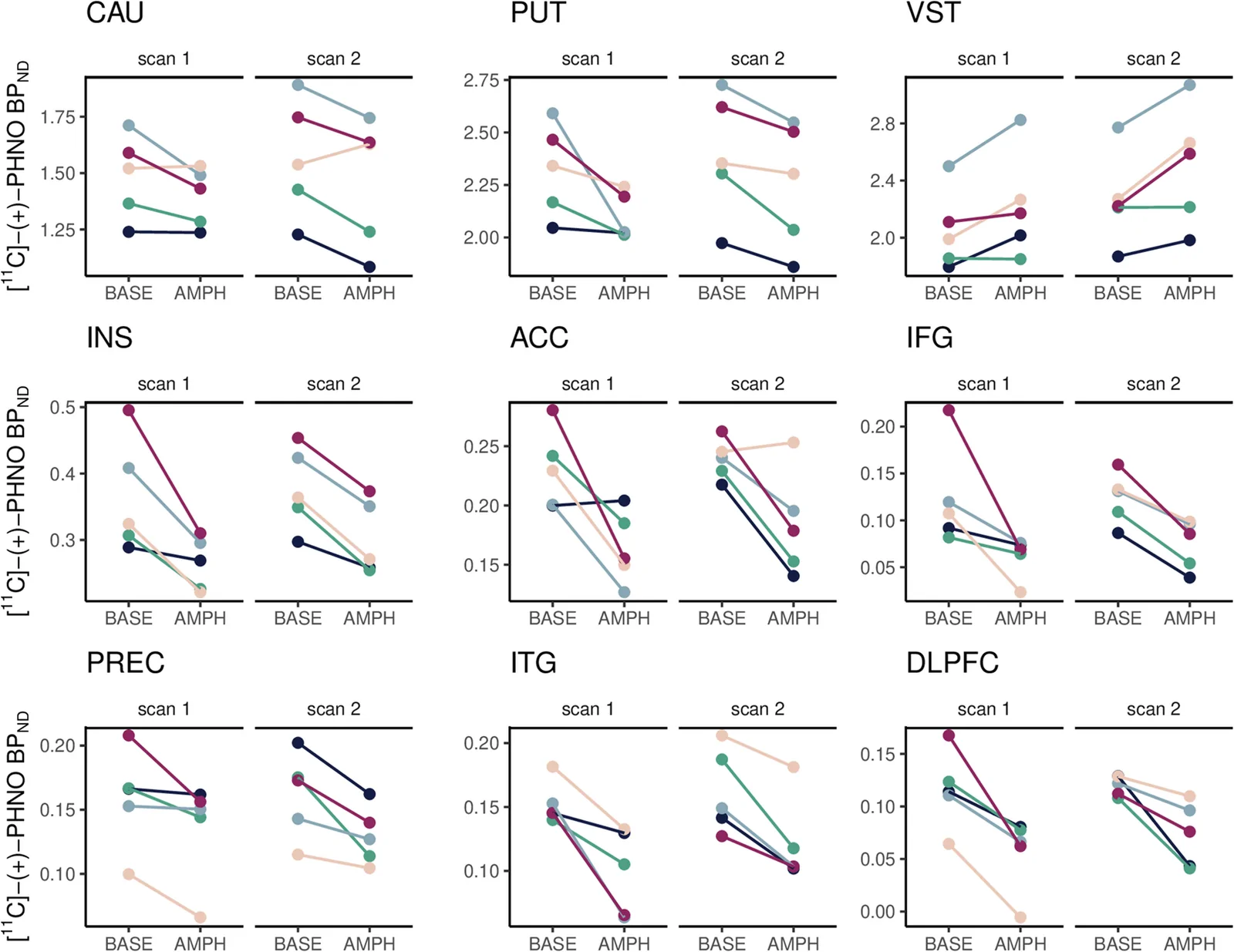
[^11^C]-(+)-PHNO BP_ND_ values before (BASE) and after intravenous amphetamine (AMPH) in selected subcortical (top row) and cortical brain regions (middle and bottom rows) CAU: caudate; PUT: putamen; VST: ventral striatum; INS: insula; ACC: anterior cingulate cortex; IFG: inferior frontal gyrus; PREC: precuneus; ITG: inferior temporal gyrus; DLPFC: dorsolateral prefrontal cortex; color-coded by volunteer

Post-amphetamine changes in BP_ND_ values were observed both in subcortical and cortical regions (**Figs. **4 and 5). The reductions in the caudate were equivalent to those reported in our previous single bolus experiment, but changes in the putamen were less pronounced [49]. In the VST, GP, as well as in the SN/VTA, tracer uptake continued to increase, although at a slower pace (**Table **S2), even after amphetamine injection, and post-amphetamine BP_ND_ values were consequently higher than at baseline. No amphetamine effects could be detected in the thalamus. In the hippocampus and the amygdala, BP_ND_ reductions were on average substantial but displayed high inter-subject variability. Among cortical ROIs, amphetamine administration was followed by a BP_ND_ decrease of 20–30% in the ACC, insula, OFC, and STG; the decrease was slightly greater in the inferior and middle temporal gyri, and reached 44% in the IFG, and 55.6% in the DLPFC.

In the second scan, amphetamine effects in all regions apart from the caudate, thalamus, amygdala, and the hippocampus met the significance threshold, but for the most part did not profoundly differ in magnitude from the BP_ND_ changes observed during the first scan. The only notable exceptions were the SN/VTA and the DLPFC, where there were pronounced differences between first and second scans (see **Table** 2). At the individual level, however, post-amphetamine measures displayed high between-scan variability, and the changes in ΔBP_ND_ were inversely proportional to first scan values (see **Table **S5 and **Fig. **S4).

## Discussion

This [^11^C]-(+)-PHNO PET study represents, to our knowledge, the first attempt at brain-wide dopamine receptor quantification using a B/I approach and a D_2/3_ receptor agonist radioligand. Combining the B/I paradigm with an i.v. amphetamine challenge allowed us to obtain single-scan estimate of baseline and post-amphetamine [^11^C]-(+)-PHNO binding without recourse to a kinetic model. This strategy proved favorable for simultaneously quantifying radioligand binding in a range of receptor-dense and receptor-sparse ROIs, but was less successful in the highest-binding regions.

With the applied bolus to infusion ratio of 80 min, near equilibrium conditions were reached in the majority of ROIs well before the amphetamine injection at 60 min, and were quickly re-established afterwards. In keeping with a previous [^11^C]-(+)-PHNO B/I study [7], baseline BP_ND_ values across the basal ganglia showed excellent test-retest reliability, comparable to estimates achieved with the single bolus approach [9, 52, 53]. Robust estimates were also obtained for several cortical areas.

Amphetamine-induced changes in [^11^C]-(+)-PHNO binding were detected in areas of frontal, temporal, and parietal cortices, as well as in parts of the striatum; post-amphetamine reductions in BP_ND_ values were, in fact, most pronounced in the prefrontal cortex, followed by the temporal cortex ROIs, consistent with the hypothesis that radioligand binding is more vulnerable to competition with endogenous dopamine in regions where baseline concentrations of the neurotransmitter are low [22, 54]. Striatal BP_ND_ decrease was at the lower end of the range described when using oral amphetamine pre-treatment and a single bolus injection of [^11^C]-(+)-PHNO [55]; in particular, changes detected in the putamen were smaller than in our previous single bolus experiment, despite comparable amphetamine doses (i.e. 0.3 mg/kg i.v. here versus 0.4 mg/kg oral in Weidenauer et al., and several other [^11^C]-(+)-PHNO studies) [9, 49, 55]. Similar observations – a uniform amphetamine challenge inducing smaller changes in receptor occupancy during B/I experiments - have been made in-head-to-head method comparison studies using [^11^C]raclopride [20] and [^18^F]fallypride [22]. Several factors could be contributing to this discrepancy. Firstly, an incomplete equilibrium before or after amphetamine injection could lead to biased BP_ND_ values, and a consequent underestimation of post-amphetamine changes in receptor binding. Secondly, due to differences in the timing of PET acquisitions relative to amphetamine administration and the scanning interval used for receptor quantification, the two methods measure amphetamine effects occurring over distinct timescales (approximately 30 min in the B/I paradigm versus up to three hours when applying the radioligand as bolus) and thus may not yield equivalent results [20]. Lastly, given that [^11^C]-(+)-PHNO is an agonist radioligand capable of inducing physiological effects even at very low doses [6, 23, 56], administering it prior to the amphetamine injection may have partly inhibited dopamine release via D_2/3_ autoreceptor-mediated effects, as seen in animal experiments employing other D_2/3_ receptor agonists [57–59].

Of note, in areas where a substantial proportion of [^11^C]-(+)-PHNO binding is to D_3_ receptors - i.e. the GP, VST, and the SN/VTA [48, 60, 61], radioactivity levels remained stable or continued to increase after amphetamine injection. Based on the previously reported high rates of change in VST and GP radioactivity measured throughout a 90 min period [7], the continued increase that we observed in D_3_-rich regions might be entirely due to unmet equilibrium conditions. Under this interpretation, amphetamine administration would have increased competition for available receptors before equilibrium had been reached and thus merely slowed radioligand accumulation in these ROIs, which would align with the decrease in the slope of the TACs seen after amphetamine injection. This hypothesis would also be consistent with prior findings of quasi-irreversible [^11^C]-(+)-PHNO binding in the GP, attributed to the high density of dopamine D_3_ receptors in this region [62]. However, a contribution of other factors cannot be excluded at this point, and the matter will require further examination in a larger sample. A systematic comparison between different modes of tracer and amphetamine administration, as well as between quantification methods [63] will be needed to disentangle the lack of equilibrium from challenge and possible ligand effects.

Insufficient proximity to equilibrium in D_3_-rich regions does not only complicate the quantification of amphetamine effects but also has implications for baseline assessments, as [^11^C]-(+)-PHNO BP_ND_ values estimated in this study likely underestimate true specific binding. Considering the rates of change observed during the baseline equilibrium time windows, the magnitude of the discrepancy should fall between 5 and 15% [7]. Obtaining a more precise estimate of this bias, or optimizing the infusion protocol will be crucial for improving accuracy, but given the high consistency in baseline measurements, this should not present an insurmountable obstacle to acquiring viable estimates in these regions. To this end, future studies should not overlook potential effects of ligand mass on [^11^C]-(+)-PHNO binding. Although the small number of volunteers and low intraindividual variability in injected mass prevented us from reliably quantifying its impact in this sample (but see **Fig. **S4 for a visual assessment), simulations performed in an earlier study indicate a substantial underestimation of receptor occupancy is possible with the mass range administered in this sample [10].

Quantifying [^11^C]-(+)-PHNO binding outside of D_2/3_ receptor dense areas was facilitated by the uptake-based thresholding of the reference region. The possibility of [^11^C]-(+)-PHNO binding to dopamine receptors in the cerebellum has been acknowledged since the initial kinetic modelling study [64], which attributed a potential BP_ND_ bias of 10–13% to a second, kinetically distinguishable compartment in the cerebellum. Subsequent experiments in humans and nonhuman animals reported dose-dependent decreases in cerebellar radioligand uptake in response to D_3_ receptor antagonists and cold radioligand [48, 65–67] supporting the existence of a specific binding compartment in this region. Similar observations have been made with other D_2/3_ radioligands across several types of pharmacological interventions [36, 37, 68, 69]. Given our interest in quantifying [^11^C]-(+)-PHNO binding in areas with low receptor densities, a small bias deriving from specific binding in the cerebellum was a bigger concern than it is for studies focusing solely on high binding areas. After reviewing published attempts to limit the impact of potential cerebellar binding in D_2/3_ receptor quantification studies, as well as strategies developed for radioligands that lack valid, anatomically segregated reference regions, we opted for a middle ground of imposing an uptake-based restriction on voxels used to estimate nondisplaceable binding. This allowed us to maximize consistency with previous [^11^C]-(+)-PHNO studies, all of which used cerebellar gray matter as the reference tissue, and reduce the likelihood of incorporating a specific binding component while retaining approximately 50% of the initial region volume. Applying an anatomically unbiased criterion that excluded high uptake voxels yielded a reference region that was spatially consistent with existing research on [^11^C]-(+)-PHNO binding in the cerebellum [48] and, importantly, failed to show strong amphetamine effects that were observed in the cerebellar cortex as a whole. While this solution requires further evaluation to determine whether a specific binding component was adequately circumvented, it is a promising and easily implemented strategy that could also support non-invasive quantification of dopamine receptors with radioligands other than [^11^C]-(+)-PHNO. The possibility of a nonspecific component, such as vascular outcomes of amphetamine administration [70], contributing to the observed changes in [^11^C]-(+)-PHNO binding will likewise need to be more fully addressed in future studies. Nevertheless, considering the magnitude of the amphetamine effects seen in this study, as well as the overall anatomical agreement with studies that performed interventions targeting dopamine receptors more specifically, we deem any such effect unlikely to have strongly influenced our results.

In summary, the data presented here indicate that, within certain constraints, synchronous measures of both baseline D_2/3_ receptor occupancy and dopamine release across a substantial part of the brain can be achieved using the B/I approach and [^11^C]-(+)-PHNO PET. While further optimization of the infusion and scanning protocols might be required to exploit the full potential of the method, these results demonstrate the feasibility of the approach which, in its current state, already offers the prospect for significantly extending our understanding of dopamine D_2/3_ mediated signaling at a systemic level.

## Supplementary information

Below is the link to the electronic supplementary material.


Supplementary File 1 (DOCX 35.6 MB)


## Data Availability

The data are available from the corresponding author upon reasonable request.
