# Supplementary material for: Continuous infusion [11C]-(+)-PHNO PET for measuring dopamine D2/3 receptor availability and amphetamine effects across the brain: Feasibility and test-retest reliability
Source: Eur J Nucl Med Mol Imaging. 2026 Jun 15;53(11):6252–63. doi: 10.1007/s00259-026-07998-w (PMC13526102; doi:10.1007/s00259-026-07998-w)
Supplement: Supplementary file 1 [file 259_2026_7998_MOESM1_ESM.docx]

# Supplementary material

**Table S1** Programs and functions used for preprocessing of PET data

| **Step** | **AFNI program** | **Source image** | **Target image** | **Cost function** | **Interpolation** |
| --- | --- | --- | --- | --- | --- |
| Motion  correction | 3dvolreg | 4D non-attenuation corrected PET  image | Mean non-attenuation corrected PET image (15-40 min) | Iterated linearized weighted least squares | Fourier (alignment) |
|  | 3dAllineate | 4D attenuation corrected PET  image |  |  | Weighted sinc (output dataset) |
| Co-registration | [a](http://afni.proc.py/)lign_epi_anat.py | 3D MPRAGE T1-weighted MRI | Mean attenuation corrected PET image (0-55 min) | Normalized mutual information | Quintic (alignment)  Weighted sinc (output dataset) |
| Normalization | 3dAllineate  (linear alignment) | 3D MPRAGE T1-weighted MRI | MNI152 2009 template (1x1x1mm) | lpa+ (local Pearson absolute correlation + weighted contribution of other cost functions) | Linear (alignment), Weighted sinc (output dataset) |
|  | 3dQWarp (nonlinear alignment, smallest segment size: 25 voxels) | linearly aligned 3D MPRAGE T1-weighted MRI | MNI152 2009 template (1x1x1mm) | lpa | Cubic (alignment),  Weighted sinc (output dataset) |
|  | 3dNwarpApply | 4D attenuation-  and motion-corrected PET  image |  |  | Linear |
|  |  |  |  |  |  |
|  | **AFNI program** | **Source image** | **Target smoothness** | **Filter shape** |  |
| Smoothing | 3dBlurToFWHM | Normalized 4D  PET image | 8mm FWHM | 3D Gaussian |  |

## **Equilibrium placement**

Equilibrium time windows were determined according to the following definitions: baseline equilibrium was defined as a minimum of 3 and a maximum of 6 consecutive frames in the 30-60 min period following tracer injection; post-amphetamine equilibrium was defined as a minimum of 3 and a maximum of 7 consecutive frames in the 60-120 min period of scanning. Within these constraints, time windows were algorithmically selected based on the rates of change in measured activity (%/h) calculated for the 15% of voxels with highest mean [^11^C]-(+)-PHNO uptake. This percentage was chosen through trial and error to consistently cover the basal ganglia, midbrain, and most of cortical regions of interest, while avoiding isolated voxels where high uptake values are likely an artifact. To ensure that the algorithmic selection provided plausible equilibrium time windows for each volunteer, regional TACs were visually inspected, and the window was shifted to the next best algorithmic candidate if necessary. In the case of the scan which was terminated early, the candidate period for post-amphetamine equilibrium was shorter (60-100min), but the procedure was otherwise the same.

The resulting equilibrium window placement for each subject and scan is depicted in **Fig. S1**. Baseline equilibrium timing was consistent across all subjects, with a nearly perfect within-subject overlap. The post-amphetamine windows were more varied across subjects, but largely consistent across scans.

**Fig. S1** Equilibrium time window placement for each volunteer and scan

**Table S2** Regional rates of change (%/h) in measured radioactivity during the pre- (BASE) and post-amphetamine equilibrium time windows (AMPH)

|  | Scan 1 |  | Scan 2 |  |
| --- | --- | --- | --- | --- |
| Region | BASE rate | AMPH rate | BASE rate | AMPH rate |
| Caudate | 3.93 ± 6.2 | -2.84 ± 6.6 | -2.07 ± 9.6 | -3.14 ± 8 |
| Putamen | 2.33 ± 9.4 | -11.5 ± 6.1 | -7.31 ± 14.3 | -11.25 ± 9.2 |
| Globus pallidus | 25.18 ± 3.5 | 4.56 ± 7.5 | 13.1 ± 12.3 | 3.76 ± 18.7 |
| Ventral striatum | 14.56 ± 11.6 | 6.55 ± 10.5 | 10.43 ± 9.8 | 7.95 ± 20.6 |
| Thalamus | -0.47 ± 8.6 | -8.82 ± 20.1 | -4.96 ± 15.1 | -12.93 ± 13.4 |
| Insula | -7.73 ± 7.9 | 5.22 ± 17 | -11.05 ± 16.7 | -5.26 ± 10.8 |
| Anterior cingulate cortex | -3.48 ± 8.5 | 1.94 ± 25.2 | -1.15 ± 12.2 | 9.69 ± 19.4 |
| Hippocampus | -1.98 ± 13.4 | -4.85 ± 17.5 | 3.17 ± 25.3 | -15.35 ± 15.6 |
| Amygdala | 0.06 ± 14 | -5.22 ± 23.8 | 5.03 ± 20.4 | -28.61 ± 22.6 |
| Substantia nigra/ Ventral tegmental area | 13.39 ± 10.5 | -0.75 ± 22.16 | 19.43 ± 34.2 | -0.04 ± 34.4 |
| Precuneus | -0.46 ± 6.7 | 8.84 ± 17.1 | -3.87 ± 11.2 | -8.96 ± 16.1 |
| Inferior frontal gyrus | -7.35 ± 8.2 | 2.01 ± 18.2 | -7.98 ± 8.8 | 10.15 ± 11.8 |
| Dorsolateral prefrontal cortex | -2.23 ± 4.9 | -4.39 ± 11.6 | -9.32 ± 9.7 | 0.59 ± 6.3 |
| Superior temporal gyrus | -8.41 ± 6.3 | -11.06 ± 7.4 | -10.03 ± 15.6 | -6.07 ± 3.2 |
| Inferior temporal gyrus | -11.11 ± 8.6 | -2.4 ± 20.1 | -9.93 ± 8.6 | -0.22 ± 8.3 |
| Middle temporal gyrus | -6.85 ± 5.1 | -12.65 ± 5.6 | -21.85 ± 14.7 | -7.4 ± 6.5 |
| Orbitofrontal cortex | -0.63 ± 7.6 | 1.65 ± 13 | -4.9 ± 14.4 | 3.47 ± 7.8 |

## **Reference region thresholding**

Thresholding was performed based on mean [^11^C]-(+)-PHNO uptake during the pre-amphetamine equilibrium. In a first step, a fine grained parcellation of the cerebellar cortex [1] was used to create an initial region mask. Before removing the vermis and other midline structures, 5% of the voxels with the lowest uptake were excluded from the mask in order to minimize the effects of residual anatomical mismatch between individual brains and the cerebellar atlas. Of the remaining voxels, 55% with the lowest uptake were retained. The uptake-based thresholding was performed for each volunteer and scan separately. Between-subject overlap in resulting reference region masks is illustrated in **Fig. S2**. Further restrictions of the masks to between-subject or between-scans overlap areas yielded virtually identical SUVs as the larger, subject- and scan-specific masks.

**Fig. S2** Between-subject overlap in reference regions masks created for each volunteer by uptake-based thresholding of the cerebellar cortex (55% of the voxels with lowest uptake at equilibrium retained). Overlap was estimated for first-scan reference region masks prior to smoothing. The color scale indicates the number of subjects (0-5) with the given voxel included in the mask.

MNI: Montreal Neurologic Institute coordinates

**Table S3** A comparison between baseline BP_ND_ values estimated in this study, and those from a previous [^11^C]-(+)-PHNO experiment performed on the same PET/CT scanning system and employing the single bolus injection method [2].

| Region* | Present study | Weidenauer et al (2020) | % difference |  |
| --- | --- | --- | --- | --- |
|  |  |  |  |  |
| Caudate | 1.52 ± 0.22 | 1.38 ± 0.23 | 9.65 |  |
| Putamen | 2.36 ± 0.25 | 2.19 ± 0.21 | 7.47 |  |
| Ventral Striatum | 2.16 ± 0.31 | 2.29 ± 0.28 | 5.84 |  |
| Globus Pallidus | 2.02 ± 0.16 | 2.11 ± 0.43 | 4.36 |  |
| Substantia nigra/  Ventral tegmental area | 0.87 ± 0.13 | 0.97 ± 0.26 | 10.87 |  |
|  |  |  |  |  |
| N volunteers (n scans) | 5 (10) | 27 (27) |  |  |
| [^11^C]-(+)-PHNO application | B/I | Single bolus injection |  |  |
| BP_ND_ quantification | equilibrium ratio | SRTM2 [3] |  |  |

*The two studies employed different methods for ROI delineation: this study used Freesurfer 6.0 software and two different anatomical atlases (see “Regions of interest” in the main manuscript for details); the Weidenauer et al. study [2] used manual delineation for Globus Pallidus and Substantia nigra/Ventral tegmental area and the automated image analysis software ROMI [4] for the other ROIs.

**Fig. S3** Baseline [^11^C]-(+)-PHNO BP_ND_ values in low-binding brain regions (color scale is adjusted to highlight differences in low BP_ND_ values)

**Table S4** [^11^C]-(+)-PHNO BP_ND_ values and test-retest estimates for Destrieux and HCP atlas regions used to create composite ROIs

| Region | Scan 1 |  | Scan 2 |  | Test-retest (BP_ND-base)_ | |  |
| --- | --- | --- | --- | --- | --- | --- | --- |
|  | BP_ND-base_ | BP_ND-amph_ | BP_ND-base_ | BP_ND-amph_ | ICC | TRV (%) | aTRV (%) |
| **DLPFC** |  |  |  |  |  |  |  |
| G_front_middle | 0.06 ± 0.05 | 0.01 ± 0.05 | 0.06 ± 0.02 | 0.03 ± 0.03 | 0.09 | -45.6 ± 148.5 | 87.48 ± 122.9 |
| S_front_inf | 0.19 ± 0.02 | 0.12 ± 0.02 | 0.19 ± 0.02 | 0.13 ± 0.05 | 0.16 | -1.87 ± 14.58 | 12.06 ± 5.9 |
| S_front_middle | 0.23± 0.02 | 0.15 ± 0.03 | 0.24± 0.02 | 0.16 ± 0.03 | 0 | -4.79± 17.8 | 15.32 ± 7.3 |
|  |  |  |  |  |  |  |  |
| **OFC** |  |  |  |  |  |  |  |
| G_orbital | 0.05 ± 0.03 | 0.01 ± 0.02 | 0.08 ± 0.04 | 0.04± 0.02 | 0.01 | -31.56 ± 75.48 | 62.94 ± 44.6 |
| G_rectus | 0.08 ± 0.04 | 0.09 ± 0.13 | 0.07 ± 0.08 | 0.09 ± 0.08 | 0.79 | -26.69 ± 96.3 | 6.24 ± 100.6 |
| S_orbital_lateral | 0.27± 0.7 | 0.18 ± 0.03 | 0.28± 0.3 | 0.2 ± 0.06 | 0.33 | -6.38 ± 26.1 | 19 ± 16.7 |
| S_orbital_med_olfact | 0.32± 0.7 | 0.33 ± 0.04 | 0.36 ± 0.05 | 0.35 ± 0.08 | 0.7 | -12.09 ± 17.2 | 14.1 ± 15.2 |
| S_orbital_H_Shaped | 0.16 ± 0.03 | 0.08 ± 0.04 | 0.17 ± 0.02 | 0.11± 0.7 | 0.57 | -6.96± 16.9 | 14.24 ± 9.7 |
| S_suborbital | 0.23± 0.02 | 0.23± 0.08 | 0.22± 0.03 | 0.21± 0.02 | 0.6 | 1.55± 10.8 | 8.47±5 |
|  |  |  |  |  |  |  |  |
| **IFG** |  |  |  |  |  |  |  |
| G_front_inf_Opercular | 0.16 ± 0.05 | 0.1 ± 0.01 | 0.17± 0.02 | 0.1± 0.02 | 0.58 | -10.19 ± 22.3 | 20.93 ± 9.1 |
| G_front_inf_Orbital | 0.12 ± 0.06 | 0.03± 0.05 | 0.1 ± 0.05 | 0.07 ± 0.05 | 0.9 | 11.7± 27.6 | 24.68 ± 13 |
| G_front_inf_Triangul | 0.08 ± 0.05 | 0.02± 0.04 | 0.07± 0.02 | 0.04± 0.05 | 0.67 | 3.27± 39.3 | 31.81 ± 17.1 |
|  |  |  |  |  |  |  |  |
| **Insula** |  |  |  |  |  |  |  |
| G_Ins_lg_and_S_cent_ins | 0.46± 0.15 | 0.3 ± 0.06 | 0.48± 0.12 | 0.35± 0.08 | 0.93 | -5.78 ± 9.8 | 9.04 ± 6 |
| G_insular_short | 0.34± 0.1 | 0.25 ± 0.08 | 0.34± 0.8 | 0.25± 0.08 | 0.92 | -0.55 ± 13.9 | 11.27 ± 6 |
| S_circular_insula_ant | 0.33± 0.1 | 0.24 ± 0.04 | 0.34± 0.7 | 0.25± 0.08 | 0.93 | -4.5± 10.7 | 9.95 ± 4 |
| S_circular_insula_inf | 0.36± 0.09 | 0.25 ± 0.03 | 0.37± 0.7 | 0.3± 0.05 | 0.85 | -3.43± 11.1 | 9.32 ± 5.5 |
| S_circular_insula_sup | 0.34± 0.7 | 0.26 ± 0.04 | 0.37± 0.3 | 0.3± 0.05 | 0.54 | -8.7± 14.5 | 12.16 ± 10.9 |
|  |  |  |  |  |  |  |  |
| **STG** |  |  |  |  |  |  |  |
| G_temp_sup_G_T_transv | 0.2± 0.04 | 0.16 ± 0.06 | 0.2± 0.01 | 0.16 ± 0.02 | 0.12 | -1.5 ± 16.8 | 12.8 ± 9 |
| G_temp_sup_Lateral | 0.11 ± 0.02 | 0.07± 0.03 | 0.13 ± 0.02 | 0.09 ± 0.02 | 0.22 | -16.59 ± 25.5 | 24.47 ± 11.8 |
| G_temp_sup_Plan_polare | 0.15 ± 0.06 | 0.1 ± 0.02 | 0.15 ± 0.04 | 0.1 ± 0.08 | 0.87 | -10.37 ± 29.3 | 19.16 ± 23.1 |
| G_temp_sup_Plan_  temporale | 0.23± 0.02 | 0.18± 0.03 | 0.24± 0.03 | 0.17± 0.03 | 0 | -4.11 ± 19.3 | 14.85 ± 10.9 |
|  |  |  |  |  |  |  |  |
| **SN/VTA** |  |  |  |  |  |  |  |
| Substantia nigra pars  compacta | 0.93 ± 0.16 | 1.12 ± 0.15 | 0.96± 0.14 | 1.11± 0.21 | 0.92 | -3.93 ± 6.18 | 15.89 ± 17.8 |
| Substantia nigra  pars reticulata | 0.78 ± 0.15 | 1.04 ± 0.15 | 0.87 ± 0.11 | 1.04 ± 0.14 | 0.91 | -11.02 ± 6.9 | 16.81 ± 10.34 |
| Ventral tegmental area | 0.94 ± 0.17 | 1.09± 0.21 | 1.01± 0.12 | 1.15± 0.29 | 0.79 | -7.69 ± 11.32 | 17.04 ± 17.5 |

## **Between-scan differences in amphetamine effects**

Test-retest metrics for post-amphetamine [^11^C]-(+)-PHNO BP_ND_ are listed in **Table S5** below. In the case of ΔBP_ND_, ICC values were below 0.5 for all regions except the thalamus, and absolute test-retest variability was in excess of 30%. The direction and magnitude of between-scan changes in ΔBP_ND_ were highly dependent on the values estimated during the first scan: lower initial ΔBP_ND_ values were associated with increased ΔBP_ND_ in the second scan and higher first-scan ΔBP_ND_ values decreased upon repeated intervention. This was most apparent in cortical ROIs, as displayed in **Fig. S4**. When assessed across all volunteers and all regions that reached equilibrium, the magnitude of first-scan ΔBP_ND_ accounted for 70% of the variance in subsequent changes (ΔBP_ND_ β=-1.06 , SE=0.07, 95%CI [-1.21, -0.89], t=-15.35; mixed linear model fitted using maximum likelihood, random slopes for volunteers and regions).

**Table S5** Test-retest metrics for post-amphetamine [^11^C]-(+)-PHNO BP_ND_

|  | **Test - retest (BP_ND-AMPH_)** | | |
| --- | --- | --- | --- |
| *Subcortical regions* | ICC | TRV (%) | aTRV (%) |
|  |  |  |  |
| CAU | 0.71 | -3.69 ± 12 | 10.36 ± 5.2 |
| PUT | 0.29 | -6.31 ± 12 | 9.63 ± 8.8 |
| VST^NE^ | 0.89 | -11.64 ± 8.4 | 12.3 ± 7.1 |
| GP^NE^ | 0.77 | -1.76 ± 8.9 | 6.72 ± 5.2 |
| SN/VTA^NE^ | 0.66 | -0.37 ±15.3 | 9.69 ± 10.8 |
| THA | 0.51 | -0.52 ± 12.3 | 9.22 ± 6.8 |
| AMY | 0.52 | -3.86 ± 34 | 25.14 ± 19.6 |
|  |  |  |  |
| *Cortical regions* |  |  |  |
|  |  |  |  |
| INS | 0.81 | -12.69 ± 9.9 | 9.88 ± 14.3 |
| ACC | 0 | -10.36 ± 38.1 | 38.14 ± 32.7 |
| HIP | 0.25 | -9.46 ± 39.1 | 22.9 ± 31.3 |
| ITG | 0.58 | -22.18 ± 29.6 | 31.8 ± 15.1 |
| MTG | 0.12 | -19.83 ± 38.2 | 36.6 ± 16.7 |
| STG | 0 | -12.68 ± 41.8 | 31.93 ± 26 |
| PREC | 0.68 | 1.22 ± 27.3 | 19.33 ±16.7 |
| IFG | 0 | -17.8 ± 68.4 | 49.34 ± 45 |
| OFC | 0 | -28.1 ± 44.5 | 43.57 ± 24.3 |
| DLPFC | 0 | -31.28 ± 115.4 | 79.94 ± 80.9 |

**Fig. S4** Between-scan changes in [^11^C]-(+)-PHNO ΔBP_ND_ values across all assessed cortical regions (color-coded by volunteer)

**Fig. S5** Baseline and post-amphetamine [^11^C]-(+)-PHNO BP_ND_ values in dopamine D_3_ receptor-rich regions color-coded by injected ligand mass

## Supplementary references

1. Diedrichsen J, Balsters JH, Flavell, et al. A probabilistic MR atlas of the human cerebellum. Neuroimage. 2009 May 15;46(1):39-46. <https://doi.org/10.1016/j.neuroimage.2009.01.045>.
2. Weidenauer A, Bauer M, Sauerzopf U, et al. On the relationship of first-episode psychosis to the amphetamine-sensitized state: a dopamine D_2/3_ receptor agonist radioligand study. Transl Psychiatry. 2020 Jan 8;10(1):2. <https://doi.org/10.1038/s41398-019-0681-5>.
3. Wu Y, Carson RE. Noise reduction in the simplified reference tissue model for neuroreceptor functional imaging. J Cereb Blood Flow Metab. 2002 Dec;22(12):1440-52. <https://doi.org/10.1097/01.WCB.0000033967.83623.34>.
4. Rusjan P, Mamo D, Ginovart N, et al. An automated method for the extraction of regional data from PET images. Psychiatry Res. 2006 Jun 30;147(1):79-89. <https://doi.org/10.1016/j.pscychresns.2006.01.011>
